# Stigmatizing attitudes towards individuals with anorexia nervosa: an investigation of attribution theory

**DOI:** 10.1186/2050-2974-1-5

**Published:** 2013-02-05

**Authors:** Kristy Zwickert, Elizabeth Rieger

**Affiliations:** 1 Research School of Psychology (Building 39), Australian National University, 0200, Canberra, ACT Australia

**Keywords:** Stigmatization, Attribution theory, Blameworthy, Anorexia nervosa, Eating disorders

## Abstract

**Background:**

Guided by Attribution Theory, this study assessed stigmatizing attitudes towards an individual with anorexia nervosa (AN) compared to obesity and skin cancer, and examined the extent to which manipulating a target individual’s level of blameworthiness affects levels of stigmatizing attitudes. One hundred and thirty-five female undergraduate students were randomly assigned to one of three conditions. Before and after receiving blameworthy or non-blameworthy information relating to the target’s condition, participants completed a series of self-report inventories measuring their emotional reactions, desire for social distance, and causal attributions regarding the target.

**Results:**

Participants reported a significantly greater desire for social distance from the target with AN compared to targets with obesity or skin cancer, and yet (contrary to Attribution Theory) attributed less blame to the target with AN. There were significant increases in stigmatization towards targets described as blameworthy relative to targets described as non-blameworthy.

**Conclusion:**

The findings provide insight into the elevated levels of stigmatizing attitudes held towards individuals with AN, and the role of Attribution Theory in partially accounting for this stigma.

## Background

Research investigating attitudes towards mental illness indicates that stigmatization is widespread [1-3]. Until recently, mental illness stigma research has focused primarily on conditions such as schizophrenia and depression, to the neglect of other psychological disorders [1]. In particular, stigma towards individuals with anorexia nervosa (AN) has received little empirical attention.

Anorexia nervosa is primarily characterized by a relentless pursuit of thinness resulting in weight loss substantially below a normal body weight [4] that predominantly affects young women [5], with a lifetime prevalence rate of approximately 1.9% and an additional 2.4% who partially meet the criteria for the disorder [6]. The disorder is often associated with a poor outcome, including a significantly elevated mortality rate, with a recent meta-analysis providing an estimate of 5.10 deaths per 1000 person-years [7]. Investigating the prevalence, characteristics, and triggers of stigmatizing attitudes towards AN is therefore an important area of research as stigma may impact on outcome. For instance, stigma has been shown to act as a barrier to accessing treatment in individuals with other forms of eating disorder psychopathology (e.g., bulimia nervosa, binge eating disorder, and eating disorder not otherwise specified) [8,9] and may also prolong the recovery process and increase the chance of relapse [10].

Attribution Theory [11] has been widely investigated in an attempt to understand the bases of stigmatizing attitudes held towards those with a mental illness. This theory asserts that when an individual’s illness is attributed to forces within his/her control, the person is likely to be held responsible for their condition, and subsequently stigmatized [12]. A number of studies informed by Attribution Theory have documented the existence of a lay perception that mental illnesses are more personally controllable than medical conditions [13] and hence may attract higher levels of stigmatizing attitudes. Medical conditions (e.g., cancer) are more likely to be perceived as onset-uncontrollable, and elicit pity and a willingness to help, whereas psychological disorders are often perceived as onset-controllable, and elicit anger and neglect [11].

Despite the scarcity of research in the area, preliminary evidence suggests that stigmatization of individuals with AN does exist [14,15]. These stigmatizing attitudes have been found to be prevalent among the general public [16], medical and nursing staff [17], and university students [18]. In accordance with Attribution Theory, individuals with AN are often perceived as being responsible for the onset of their illness and as having a significant amount of control over their eating disordered behaviours [19,20]. Such attributions of controllability have been identified in two college-aged samples. In the first, approximately two-thirds of female respondents agreed that individuals with AN only have themselves to blame for their condition [21]. In the second mixed-gender sample, attributing greater levels of responsibility for the development of AN was associated with higher stigmatizing attitudes towards the individual with the condition [22]. It has also been suggested that individuals exposed to sociocultural versus medical causal accounts are more likely to hold individuals with AN responsible for their condition [23] and demonstrate less willingness to provide assistance to individuals with AN [24]. However, research to date on Attribution Theory and stigma in the context of AN is limited by the preponderance of correlational methodologies, with experimental paradigms needed to provide more definitive support for the role of blame-based accounts in causing stigmatizing attitudes.

In contrast to the paucity of research on AN, stigma towards another weight condition – namely, obesity – has received a significant amount of research attention [25]. Obesity stigmatization has been described as extremely pervasive to the extent that, “negative attitudes towards obese people constitute one of the last socially acceptable forms of discrimination” [26]. The rising prevalence of obesity does not appear to have attenuated stigmatizing attitudes towards obese individuals. In fact, these attitudes may be on the increase [27].

Preliminary research suggests that levels of stigma may be equivalent towards individuals with AN and obesity. Specifically, an early study found that participants of high school and university age demonstrated comparable levels of rejection towards individuals with AN and obese individuals (although no significance testing was undertaken) [15]. As such, the obesity literature may be useful in advancing understandings of AN stigma. Stigmatization towards obese individuals draws further parallels with AN stigma in that negative attitudes occur in the context of beliefs about controllability and personal responsibility (i.e., for excess body weight) [25]. For instance, among a sample of respondents from six nations, attributions of controllability (together with holding a negative view of fatness) predicted stigmatizing attitudes towards obese people [28]. Given that perceptions of blameworthiness and personal control appear to be common towards both obesity and AN, it seems appropriate to draw on the expansive body of obesity stigma literature to more clearly define the levels and potential causes of stigmatizing attitudes held towards individuals with AN.

The present study aimed to investigate (i) the relative level of stigma towards individuals with AN and (ii) primarily through the use of an experimental design, the factors that may account for the existence of stigmatizing attitudes. Obesity was selected as a point of comparison as it is a weight condition with a substantial research base of documented negative attitudes and behaviours towards obese individuals. Skin cancer was selected as a control group against which to identify elevated levels of stigma towards individuals with AN given that medical conditions have been found to be associated with lower levels of stigma compared to psychological disorders. For example, participants in one community-based study reported a greater willingness to interact with an individual described as having skin cancer compared to an individual described as having schizophrenia or major depression [29]. Skin cancer also lends itself to a blame-based manipulation given that behavioural factors (such as sun exposure) are implicated in the etiology of the condition.

In line with Attribution Theory, it was hypothesized that AN (a mental illness) would be associated with significantly higher levels of blame and stigmatizing attitudes compared to skin cancer (a medical condition) and comparable levels to that of obesity (a highly stigmatized weight condition). Additionally, and also in accordance with Attribution Theory, it was hypothesized that, after reading a causal explanation of the condition, participants exposed to blame-based accounts would report greater increases in levels of stigma compared to participants exposed to non-blame-based causal accounts irrespective of the type of condition (i.e., AN, obesity or skin cancer).

## Methods

### Participants

One hundred and fifty-two female undergraduate students from the Australian National University (ANU) were recruited through flyers posted on campus. AN predominantly affects young women between 16 and 24 years of age [5]. Thus, the inclusion criteria required participants to be 24 years or younger as the attitudes held by peers of individuals with AN were the primary focus of this study. Seventeen participants were excluded for incomplete responses or exceeding the age criterion. The final sample consisted of 135 participants, with a mean age of 19.78 years (*SD* = 1.27; Range = 18–23). Participants self-identified as Caucasian (58.6%) or Asian (41.4%). These figures approximate the composition of the Australian population, in that the two largest ethnic groups are represented [30]. The study received ethical approval from the ANU’s Human Research Ethics Committee and informed consent was obtained from all participants.

### Design

The study employed a 3 (AN, obesity, or skin cancer) × 2 (blameworthy versus non-blameworthy) × 2 (time 1 versus time 2) factorial design. The dependent variables consisted of stigmatizing attitudes (i.e., emotional reactions and desire for social distance) in regards to the target individual and causal attributions regarding the target’s condition.

### Measures

Participants completed the Affective Reaction Scale (ARS) [31] to assess their emotional reactions towards the target individual. The ARS consists of 10 bipolar items (e.g., apprehensive versus comfortable), with higher scores indicating more positive emotional reactions. Cronbach’s alpha for the ARS in the present sample was .82.

Social Distance Scales (SDS) are commonly used in stigma research as a proxy measure of behavioural stigmatization towards individuals with a mental illness [32]. Participants completed the SDS [31] to assess their willingness to have social contact (e.g., live in a share house) with the target individual. An additional item was included to measure participants’ willingness to engage in more intimate social interactions with the target (i.e., have as a close personal friend). Each of the eight items was presented on a four-point Likert scale (where 0 = ‘definitely unwilling’ and 3 = ‘definitely willing’), with higher scores indicating a greater willingness to have social contact with the target individual. Cronbach’s alpha for the SDS in the present study was .92.

To assess causal attributions, four items relating to genetic factors, social influences, parental influences, and personal choice [33] were each presented on a seven-point Likert scale (ranging from 1 = ‘did not contribute at all’ to 7 = ‘was the main causal factor’). Participants were asked to “rate the extent to which you think the following factors contributed to the development of (the condition) in Kelly”. A fifth item (also presented on a seven-point Likert scale) measured the extent to which participants perceived the target to be blameworthy for her condition.

### Materials

Two vignettes (A and B) were developed for the AN, obesity, and skin cancer conditions. The information contained in each vignette is outlined in the Appendix. Vignette A outlined the target individual’s medical, cognitive, social, and emotional problems. These problems were selected so as to be veridical representations of each condition while also being comparable across conditions. Vignette B either described the target as being responsible for her condition (blameworthy group) or the target’s condition as being largely due to genetic influences (non-blameworthy group). For example, the blameworthy AN vignette depicted the target as deliberately choosing to restrict her food intake and exercise excessively, ignoring support from friends and family, and disregarding advice from her doctor and dietitian. In contrast, the non-blameworthy AN vignette described the target as feeling compelled to restrict her eating, having a strong family history of the illness, actively engaging in psychotherapy, and attempting to consume larger portions of food despite intense feelings of anxiety. The vignettes were approximately 150 words in length and were designed to be comparable in their descriptions of symptom severity regardless of whether they were describing a target with AN, obesity or skin cancer.

A series of pilot studies were conducted in which the vignettes were sequentially modified based on feedback from pilot participants. Data from the final pilot study indicated that, as desired, all three versions of Vignette A were equivalent in terms of perceived severity of the condition (i.e., on a 7-point scale of severity, a score of 6.5 for the AN, 6.5 for the obesity, and 6.3 for the skin cancer vignettes). In addition, the blameworthy versions of Vignette B elicited stronger attributions of blame (i.e., on a 7-point scale of blameworthiness, a score of 4.6 for the AN, 5 for the obesity, and 6 for the skin cancer vignettes) compared to the non-blameworthy versions of Vignette B (i.e., on a 7-point scale of blameworthiness, a score of 2 for the AN, 3.5 for the obesity, and 3.9 for the skin cancer vignettes).

### Procedure

Participants were randomly assigned to one of six experimental groups: AN blameworthy; AN non-blameworthy; obesity blameworthy; obesity non-blameworthy; skin cancer blameworthy; or skin cancer non-blameworthy. Participants began by reading Vignette A that described the target’s (“Kelly’s”) symptoms (relating to AN, obesity or skin cancer). Participants then completed a series of self-report inventories assessing their attitudes towards the target in terms of levels of stigma (i.e., the Affective Reaction Scale and the Social Distance Scale) and causal attributions (i.e., genetic factors, social influences, parental influences, personal choice, and overall levels of blameworthiness). The participants were then asked to read Vignette B in which the target was described as either blameworthy or non-blameworthy for her condition. The measures assessing stigmatizing attitudes and levels of blameworthiness were then re-administered. Finally, participants provided demographic information including age, ethnicity, and country of birth. Height and weight were also collected to calculate Body Mass Index (BMI = kg/m^2^).

### Statistical analysis

Differences in age and BMI across conditions were assessed using one-way between-groups analysis of variance (ANOVA). A series of planned contrasts were undertaken to determine whether there were significant differences in stigmatizing attitudes (i.e., the ARS and SDS) and causal attributions between the AN and obesity conditions, and between the AN and skin cancer conditions after reading Vignette A. Difference scores were calculated by subtracting Time 1 scores (after reading Vignette A) from Time 2 scores (after reading Vignette B) for both the ARS and the SDS. Positive difference scores indicated that participants showed an increase in positive emotional and behavioural reactions towards the target from Time 1 to Time 2. A series of planned contrasts were then conducted to investigate the hypotheses that: (i) participants in the blameworthy groups would report significantly greater increases in stigmatizing attitudes from Time 1 (after reading Vignette A) to Time 2 (after reading Vignette B) than the non-blameworthy groups as indexed by mean difference scores on the ARS and SDS; and (ii) there would be no significant interactions between type of condition and level of blameworthiness on ARS and SDS mean difference scores. Confidence intervals were calculated for all analyses and the significance level (two-tailed) was set at *p* < 0.05.

## Results

### Sample characteristics

A one-way between-groups ANOVA revealed no significant differences between the six groups in terms of mean age (*M* = 19.78, *SD* = 1.27), *t*_(132)_ = 1.727, *p* = .182, and BMI (*M* = 21.9, *SD* = 4.75), *t*_(132)_ = 1.068, *p* = .347. As such, age and BMI were not controlled for in subsequent analyses.

### Time 1 levels of stigmatizing attitudes across conditions

Planned contrasts were used to compare Time 1 scores on the ARS and SDS across conditions. Consistent with the hypotheses, there was no significant difference in mean scores on the ARS in the AN compared to the obesity condition, *t*_(132)_ = −1.304, *p* = .194, 95% CI [−6.31, 1.19]. Yet participants reported significantly lower mean scores on the SDS in the AN compared to the obesity condition, *t*_(132)_ = −2.981, *p* = .003, 95% CI [−5.43, -1.01], indicating a greater desire for social distance from the target with AN compared to the obese target. Contrary to hypotheses, there was no significant difference in mean scores on the ARS in the AN compared to the skin cancer condition, *t*_(132)_ = −1.885, *p* = .062, 95% CI [−7.63, .33]. As expected, participants reported a significantly lower mean score on the SDS in the AN as compared to the skin cancer condition, *t*_(132)_ = −2.925, *p* = .004, 95% CI [−5.23, -.97], indicating a greater desire for social distance in the former relative to the latter. Table 1 outlines the mean scores on the ARS and SDS across the three conditions.

**Table 1:** Mean (SD) scores on causal attribution scales across the three conditions

		**Disorder**		
**Dependent variable**		**Anorexia nervosa**	**Obesity**	**Skin cancer**
		**n = 48**	**n = 42**	**n = 45**
ARS	*M*	38.96	41.52	42.60
	(*SD*)	(9.31)	(8.48)	(10.02)
SDS	*M*	13.54	16.76	16.64
	(*SD*)	(5.49)	(4.99)	(4.80)
Blame	*M*	3.33	4.48	3.27
	(*SD*)	(1.58)	(1.04)	(1.54)
Genetic influences	*M*	4.08	4.86	4.89
	(*SD*)	(1.40)	(1.20)	(1.30)
Social influences	*M*	6.15	5.48	5.07
	(*SD*)	(.92)	(1.04)	(1.37)
Parental influences	*M*	4.65	5.12	3.51
	(*SD*)	(1.34)	(1.19)	(1.77)
Personal Choice	*M*	5.04	5.64	5.42
	(*SD*)	(1.60)	(1.10)	(1.10)

### Time 1 levels of causal attributions across conditions

Contrary to expectations, planned contrasts revealed significant differences in levels of blame attributed to the AN versus obese target, with the obese target eliciting significantly higher mean blame scores compared to the AN target, *t*_(132)_ = −3.811, *p* < .001, 95% CI [−1.72, -.58]. Also unexpectedly, there was no significant difference on mean blame scores in the AN versus the skin cancer condition, *t*_(132)_ = .226, *p* = .821, 95% CI [−.60, .72]. Similar results were obtained for the degree to which the condition was attributed to personal choice. Specifically, the mean personal choice score was significantly higher for obesity as compared to AN, *t*_(132)_ = −2.191, *p* = .030, 95% CI [−1.18, -.02], but not significantly different for the AN and skin cancer targets, *t*_(132)_ = −1.413, *p* = .160, 95% CI [−.96, .20].

Mean scores for the degree to which the condition was attributed to genetic influences were significantly lower for the AN target compared to both the obese and skin cancer targets, *t*_(132)_ = −2.804, *p* = .006, 95% CI [−1.33, -.23] and *t*_(132)_ = −2.972, *p* = .004, 95% CI [−1.38, -.24] respectively. The AN target received significantly higher attributions of social influence than both the obese and skin cancer targets, *t*_(132)_ = 2.816, *p* = .006, 95% CI [.26, 1.08] and *t*_(132)_ = 4.620, *p* < .001, 95% CI [.60, 1.56]. Mean parental influence scores were not significantly different for the AN and obesity targets, *t*_(132)_ = −1.536, *p* = .127, 95% CI [−1.00, .06], but were significantly higher for the AN target compared to the skin cancer target, *t*_(132)_ = 3.751, *p* < .001, 95% CI [.49, 1.79]. Mean scores on the causal attribution scales across the three conditions are shown in Table 1.

### Changes (from Time 1 to Time 2) in levels of stigmatizing attitudes following exposure to blame-based attributions

As a manipulation check for the induction of blame-based attributions, mean difference scores for levels of blame were calculated to index the change from Time 1 (after reading Vignette A) to Time 2 (after reading Vignette B). Planned contrasts revealed that Vignette B was effective in altering perceptions of blameworthiness. That is, after reading Vignette B, participants in the blameworthy groups reported significantly greater increases in levels of blame compared to participants in the non-blameworthy groups, t_(129)_ = −8.635, *p* < .001, 95% CI [−1.95, -2.11]. There was no significant interaction on blame difference scores between levels of blameworthiness and the AN versus obesity groups, t_(129)_ = .487, *p* = .627, 95% CI [−.43, .71], nor between levels of blameworthiness and the AN versus skin cancer groups, t_(129)_ = .314, *p* = .754, 95% CI [−.47, .65]. These results suggest that the successful manipulation of blameworthiness in the blameworthy versus non-blameworthy groups was comparable across conditions. Mean blame difference scores across the six experimental groups are presented in Table 2.

**Table 2:** Mean (SD) difference scores on the blame scale, ARS and SDS across the six experimental groups

		**Blame**					
		**Blameworthy**			**Non-blameworthy**		
**Difference scores**		**AN**^**1**^	**OB**^**2**^	**SC**^**3**^	**AN**^**1**^	**OB**^**2**^	**SC**^**3**^
		**n = 26**	**n = 22**	**n = 22**	**n = 22**	**n = 20**	**n = 23**
Blame Scale	*M*	1.19	1.45	1.18	-.68	-.70	-.87
	(*SD*)	(1.27)	(1.06)	(1.50)	(1.46)	(1.08)	(1.66)
ARS	*M*	−5.50	−10.95	−2.41	6.77	7.85	3.74
	(*SD*)	(7.31)	(10.08)	(8.95)	(7.11)	(6.05)	(8.84)
SDS	*M*	−3.58	−5.91	−2.27	2.50	1.15	1.78
	(*SD*)	(2.55)	(5.18)	(4.03)	(4.61)	(1.63)	(2.64)

Positive scores indicate that participants attributed more blame to the target at Time 2 compared to Time 1.

^1^ Anorexia Nervosa.

^2^ Obesity.

^3^ Skin Cancer.

**Figure 1:**
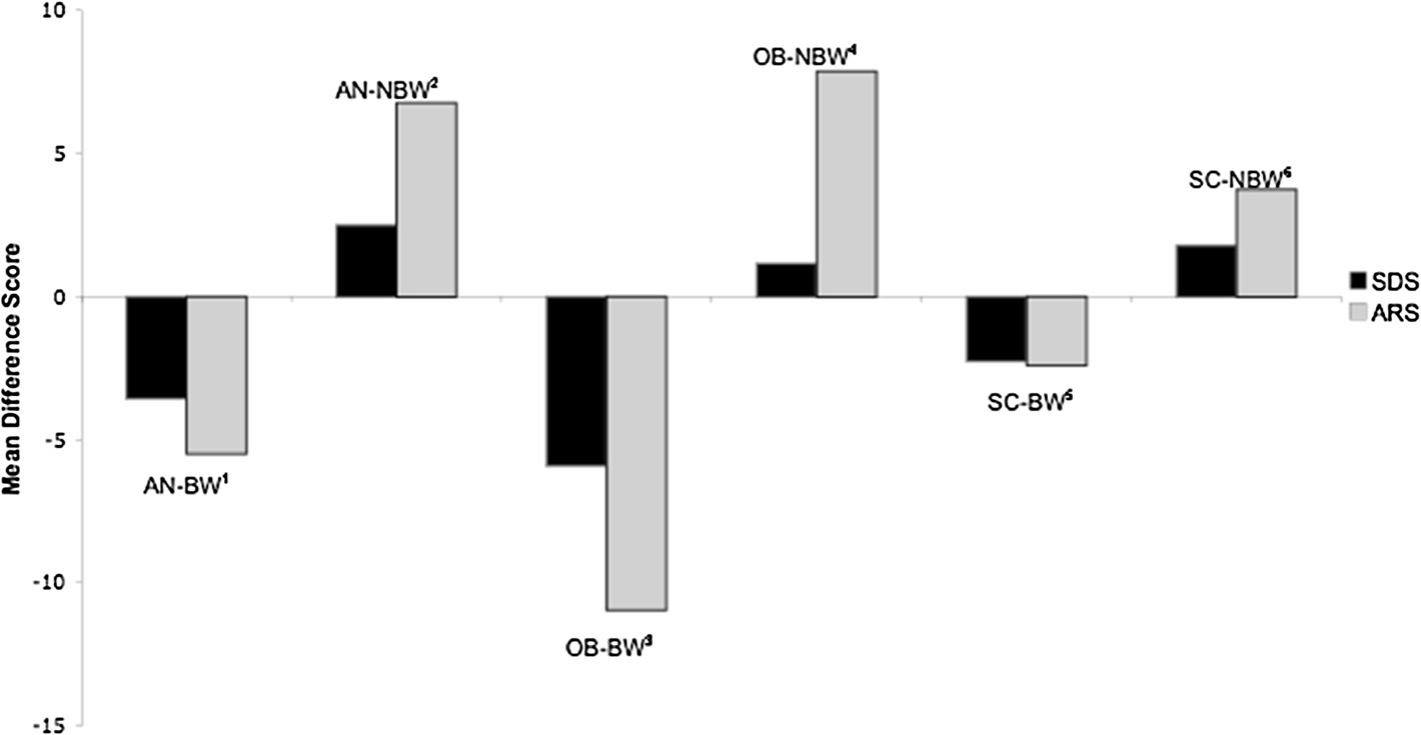
**Mean ARS and SDS Difference Scores Across the Six Experimental Groups.** AN-BW^1^ = anorexia nervosa blameworthy condition; AN-NBW^2^ = anorexia nervosa non-blameworthy condition; OB-BW^3^ = obesity blameworthy condition; OB-NBW^4^ = obesity non-blameworthy condition; SC-BW^5^ = skin cancer blameworthy condition; SC-NBW^6^ = skin cancer non-blameworthy condition.

Mean difference scores on the ARS and SDS across the six experimental groups are also shown in Table 2. As predicted, planned contrasts revealed significant differences between the blameworthy and non-blameworthy groups on mean ARS and SDS difference scores, t_(129)_ = 8.788, *p* < .001, 95% CI [11.74, 13.08] and t_(129)_ = 9.099, *p* < .001, 95% CI [5.43, 6.03] respectively. As shown in Figure 1, after reading Vignette B, participants in the blameworthy groups reported increases in stigmatizing attitudes while participants in the non-blameworthy groups reported decreases in negative attitudes towards the target.

There were no significant interactions between type of condition and level of blame for mean ARS difference scores. Specifically, there was no significant difference between the AN and obesity groups and level of blameworthiness on ARS change scores, t_(129)_ = −1.886, *p* = .062, 95% CI [−6.69, 0.16] nor between the AN and skin cancer groups and level of blameworthiness on ARS change scores, t_(129)_ = 1.001 *p* = .074, 95% CI [−0.30, 6.42]. Nor were there significant interactions between type of condition and level of blame for mean SDS difference scores. That is, there was no significant difference between the AN and obesity groups and level of blameworthiness on SDS change scores, t_(129)_ = −.636, *p* = .526, 95% CI [2.02, 1.04], nor between the AN and skin cancer groups and level of blameworthiness on SDS change scores, t_(129)_ = 1.333, *p* = .105, 95% CI [−0.49, 2.51].

## Discussion

To our knowledge, this is the first study to document levels of stigmatizing attitudes held towards individuals with AN relative to both obesity (a highly stigmatized weight disorder) and a medical condition (namely, skin cancer). Moreover, in contrast to previous correlational research, the present study’s adoption of an experimental methodology allows for the most robust examination of the causes of stigma, specifically, the effect of blame-based accounts in contributing to stigmatizing attitudes.

While it was hypothesized that stigmatizing attitudes would be held towards the target with AN, such attitudes were even stronger than anticipated. That is, participants reported comparable levels of negative affective reactions to the targets with AN and another highly stigmatized condition (i.e., obesity) yet were even more resistant to having social contact with the AN target compared to the obese target. Regarding the latter finding, participants reported that, on average, they were reluctant to associate (even indirectly) with the AN target, yet were ‘probably or definitely willing’ to have social contact with the obese target. Despite the pervasive nature of obesity stigma, these results provide evidence of even greater levels of stigmatizing attitudes towards people with AN, at least in the domain of social contact with such individuals.

The findings were partially supportive of the hypothesis that psychological conditions are more highly stigmatized than medical conditions, with the AN target attracting a significantly greater desire for social distance compared to the skin cancer target in terms of mean scores on the Social Distance Scale (SDS). Mean SDS scores indicated that while participants were somewhat reluctant to associate (even indirectly) with the AN target, they were ‘probably or definitely willing’ to have social contact with the skin cancer target. However, the difference between mean Affective Reaction Scale (ARS) scores in the AN and skin cancer groups failed to reach significance, suggesting that willingness to engage in social contact may be a more sensitive index of stigmatizing attitudes than negative emotional reactions.

The second aim of the study was to evaluate the role of blameworthy attributions in the manifestation of stigmatizing attitudes. This aim was initially addressed by investigating the types of causal attributions attributed to target individuals with AN, obesity or skin cancer after reading Vignette A. It was found that participants accorded significantly higher levels of blame and personal control to the obese target relative to the target with AN. The unexpected finding that participants attributed significantly less blame to the AN target and yet reported more stigmatizing attitudes (in terms of a desire for social distance) towards AN compared to obesity appears contrary to Attribution Theory and suggests that factors other than blame-based attributions contribute to stigma. One such factor may be personal contact with a member of a stigmatized group. In the case of the present study, AN and obesity have highly contrasting prevalence rates (approximately 1.9% for AN [6] versus 28.3% for obesity [34]). Given these differences, people are comparatively less likely to have personal contact with an individual diagnosed with AN. Research indicating that individuals who have not had contact with a person with AN are more likely to endorse stigmatizing attitudes compared to those who are familiar with a person who has AN may be one factor that accounts for why AN was more stigmatized in the current study [33]. However, given evidence that the type of contact can impact differentially on attitudes towards individuals with AN (e.g., living with a person with AN being associated with negative attitudes and watching a relevant movie or television show featuring an individual with AN being associated with more positive attitudes), caution is required in selecting contact strategies that are conducive to reducing stigma [21]. A second explanation for the attenuated ratings of blame towards individuals with AN despite elevated stigma is that some symptoms associated with the condition may be perceived as desirable [21]. For example, one study found that despite holding stigmatizing attitudes towards individuals with AN, participants reported admiring their ability to control their eating and exercise [18].

Other findings were consistent with Attribution Theory. For instance, genetic influences were perceived as significantly less relevant to AN than both obesity and skin cancer. The perceived lesser role of this non-blameworthy factor may, according to Attribution Theory, contribute to the observed higher levels of stigmatizing attitudes evident towards those with AN. Although research in the area is in its infancy (particularly in terms of the reliance on family and twin studies and an absence of adoption studies), there is some support for the role of genetic factors in the development of AN [35,36]. A lack of awareness of the possible genetic underpinnings of AN may be one of the reasons that individuals with AN are stigmatized by members of the general public [20]. Furthermore, two recent studies provide support for the dissemination of information relating to the biological underpinnings of AN in that participants exposed to such explanations demonstrated less blaming attitudes compared to participants provided with a sociocultural explanation of the condition [23,24].

In addition to being attributed less to genetic factors, the finding of significantly higher endorsement of social influences for AN can also be interpreted using Attribution Theory. Specifically, individuals with AN may be perceived as having succumbed to social pressures to be thin, with attributions of blame for ‘giving in’ to social pressures possibly translating into negative responses towards them [24].

The experimental manipulation of blame-based accounts of the three conditions provided the most methodologically robust means for evaluating the causal role of blameworthy attributions in eliciting stigmatizing attitudes. The results of the experimental manipulation were consistent with Attribution Theory’s key premise that attributions of blame result in negative affective reactions and social responses towards the target person, with the blameworthy groups experiencing significantly greater deterioration in their negative emotional reactions and desire for social distance from the target than the non-blameworthy groups. As can be seen in Figure 1, across each condition, participants in the blameworthy groups demonstrated an increase in negative emotional reactions and a desire for social distance while participants in the non-blameworthy groups demonstrated a decrease in these negative responses. There was no significant interaction between levels of blameworthiness and type of condition in terms of the desire for social distance suggesting that, irrespective of the type of condition, blame-based accounts resulted in a stronger increase in the desire for social distance from the target than non-blameworthy accounts. However, there was a trend towards significance in terms of a greater change in affective reactions in the obesity versus the AN groups, and in the AN versus skin cancer groups, when exposed to blameworthy versus non-blameworthy accounts. Given that these findings may have become significant with a larger sample, the issue as to whether blameworthy accounts may be of even greater relevance in accounting for stigma in certain conditions relative to others requires further investigation.

### Study limitations

Several limitations of the present study must be noted. First, although the sample was generally representative of the broader population in terms of ethnicity, participants were young female undergraduate students, primarily studying psychology. Thus, the current findings may not generalize to more diverse samples with regard to age, gender, and educational background. In particular, the attitudes of mothers warrant investigation as a higher level of maternal criticism has been found to be a strong predictor of poorer treatment response among adolescents with an eating disorder [37]. Moreover, the attitudes of young men may also be worthy of exploration given initial findings that some hold unsympathetic attitudes towards those with an eating disorder [38].

Second, the measures of stigmatizing attitudes used in this study were explicit rather than implicit. However, in addition to concerns about the validity and reliability of implicit measures [39], there are currently no tests available to measure implicit stigmatizing attitudes towards AN, hence the current study’s reliance on explicit measures. Additionally, levels of blame were assessed with one item given the absence of psychometrically sound instruments to assess causal attributions in the context of AN. Hence these measures need to be developed as they have been for other conditions (e.g., the Anti-Fat Attitudes Scale for obesity [40]).

Third, the present study investigated anticipated emotional and social reactions towards a fictional character with AN. As such, an important limitation of this study is that *actual* emotional and social reactions towards a person with AN were not examined. Although this is an important first step, more ecologically-valid research is needed to understand the relationship between causal attributions and responses towards stigmatized individuals [1].

Finally, the study may not have had sufficient power to detect significant interactions between level of blameworthiness and type of condition. Post-hoc analyses for the two interaction contrasts (i.e., level of blameworthiness and AN versus obesity on ARS scores, and level of blameworthiness and AN versus skin cancer on ARS scores) revealed a moderate ability to detect a medium effect size (i.e., with power of 65% and 67% respectively at a significance level of .05). Therefore, the notion that blame-based accounts result in comparable increases in stigma irrespective of the type of condition must be considered a preliminary finding given that the findings on the ARS approached significance.

### Future directions

Given the lack of research in the area of AN stigma, much remains to be deciphered. Future research should explore additional factors that potentially reduce stigmatizing attitudes towards individuals with AN. Carefully selected contact strategies such as those involving people with personal experience of the disorder may assist with disconfirming faulty beliefs regarding personal blame for the illness [41,42]. In addition, information disseminated to the community could provide nuanced explanations of AN, emphasising that it is a serious psychological condition influenced by a range of biopsychosocial factors, including genetics.

Additionally, it will be important to ascertain the impact of stigma from the perspective of individuals with AN [43] as has been conducted for other mental illnesses such as schizophrenia [44]. It is possible that the perception and experience of stigma in individuals with AN may differ from those with other psychological disorders. For instance, the fact that individuals with AN often experience the condition as egosyntonic may prove protective against the negative attitudes of others towards them [45]. Conversely, given the association between eating disorder symptomatology and rejection sensitivity [46], individuals with an eating disorder may be even more vulnerable to the negative consequences of stigmatizing attitudes.

## Conclusions

This study contributes to the small body of research examining stigmatizing attitudes held towards individuals with AN. Elevated levels of stigma towards individuals with AN were found, with some evidence (i.e., in the domain of desired social contact) that these attitudes may be even stronger than those held towards obese individuals. In addition, blame-based accounts were found to increase stigma towards an individual with AN (as well as those with obesity and skin cancer), providing support for the etiological significance of blameworthy attributions for AN stigma. In demonstrating that stigmatizing attitudes in AN can be modified via the manipulation of levels of blameworthiness, the results may help to inform interventions designed to ameliorate such attitudes.

## Appendix

Table 3 provides an overview of the information contained in each vignette.

**Table 3:** Summary of vignettes for the six experimental groups

**Vignette A**		
**Anorexia nervosa**	**Obesity**	**Skin cancer**
• 19-year-old psychology student who has AN	• 19-year-old psychology student who is obese	• 19-year-old psychology student who has melanoma
• Extremely underweight	• Extremely overweight	• Most dangerous form of skin cancer that required chemotherapy
• Very self-conscious about her body	• Very self-conscious about her body	
		• Very self-conscious about her hair loss
• Avoids social situations, especially those that require her to eat in public	• Avoids social situations, especially those that require her to eat in public	
		• Avoids social situations due to embarrassment
• Feels very sad and anxious about her body	• Feels very sad and anxious about her body	• Feels very scared and anxious about her recent health condition
• Experiences irregular heartbeats and is at high risk of heart attack	• Experiences high blood pressure and is therefore at high risk of heart attack	• At risk of the cancer returning and spreading throughout her body
**Vignette B** – **Blameworthy version**		
• Deliberately chooses to restrict eating and exercise excessively	• Deliberately chooses to overeat and avoid exercise	• Deliberately spent time in the sun to get a tan
	• Not willing to consider other ways to experience pleasure	• Not willing to consider other ways to feel good about herself
• Not willing to consider other ways to feel good about herself		
	• Chooses to focus solely on getting pleasure from eating	• Stuck to usual routine of spending time lying in the sun
• Chooses to focus solely on continuing to lose weight		
• Gets annoyed with friends who provide unwanted advice	• Gets annoyed with friends who provide unwanted advice	• Chose to ignore advice from friends who encouraged her to use sunscreen and wear a hat
• Ignores advice from doctor and dietitian	• Ignores advice from doctor and dietitian	
**Vignette B** – **Non**-**blameworthy version**		
• Driven by illness to restrict eating and exercise excessively	• Driven by strong appetite to engage in overeating	• Illness results in feeling self-conscious about hair-loss
• History of AN within her family	• History of obesity within her family	• History of cancer within her family
• Doctor has explained genetic contributions to the condition	• Doctor has explained genetic contributions to the condition	• Doctor has explained genetic contributions to the condition
• Has made many personal attempts at gaining weight	• Has made many personal attempts at losing weight	• Has made many personal attempts at being cautious in the sun
• Referred to clinical psychologist and actively participating in therapy	• Referred to clinical psychologist and actively participating in therapy	• Referred to cancer specialist and actively monitoring changes in skin
• Trying to eat more even though it makes her anxious	• Trying to walk more even though it is physically uncomfortable	• Trying to keep out of the sun as much as possible

Note. Participants received one copy of Version A corresponding with the condition to which they were assigned (i.e., AN, obesity, or skin cancer) and one copy of Version B (describing the individual as either blameworthy or non-blameworthy).

## Competing interests

The authors declare that they have no competing interests.

## Authors’ contributions

KZ and ER designed the study, conducted the statistical analysis, and drafted the manuscript. KZ carried out the data collection. Both authors read and approved the final manuscript.
